# Identifying the quality of life effects of urinary incontinence with depression in an Australian population

**DOI:** 10.1186/1471-2490-13-11

**Published:** 2013-02-16

**Authors:** Jodie C Avery, Nigel P Stocks, Paul Duggan, Annette J Braunack-Mayer, Anne W Taylor, Robert D Goldney, Alastair H MacLennan

**Affiliations:** 1 Discipline of General Practice, The University of Adelaide, Adelaide, Australia; 2 Discipline of Obstetrics and Gynaecology, The University of Adelaide, Adelaide, Australia; 3 Discipline of Public Health, The University of Adelaide, Adelaide, Australia; 4 Population Research and Outcome Studies, Discipline of Medicine, The University of Adelaide, Adelaide, Australia; 5 Discipline of Psychiatry, The University of Adelaide, Adelaide, Australia

## Abstract

**Background:**

To explore the additive effect of urinary incontinence, in people with comorbid depression, on health related quality of life.

**Methods:**

Males and females, 15 to 95 years (n = 3010, response rate 70.2%) were interviewed face to face in the 1998 Autumn South Australian Health Omnibus Survey.

**Results:**

Self-reported urinary incontinence was found in 20.3% (n=610), and depression as defined by the PRIME-MD in 15.2% (n=459) of the survey population. Urinary incontinence with comorbid depression was found in 4.3% of the overall population. Univariate analysis showed that respondents with urinary incontinence and comorbid depression were more likely to be aged between 15 and 34 years and never married when compared to those with incontinence only. Multivariate analysis demonstrated that in people with incontinence, the risk of having comorbid depression was increased by an overall health status of Fair or Poor, or the perception that their incontinence was moderately or very serious. Respondents reporting that they experienced incontinence with comorbid depression scored significantly lower than those experiencing incontinence without depression on all dimensions of the SF-36.

The interaction of the presence of incontinence and the presence of depression was significantly associated with the dimensions of physical functioning.

**Conclusions:**

Depression and incontinence both reduce QOL. When they occur together there appears to be an additive effect which affects both physical and mental health, perhaps by increasing a person’s negative perceptions of their illness. Clinicians should identify and manage comorbid depression when treating patients who have incontinence to improve their overall QOL.

## Background

Associations between urinary incontinence and depression have been found previously [1,2]. Explanations for this relationship include biochemical factors [1], or the severity of incontinence [3]. For instance in animal models, lowering monoamines such as serotonin and noradrenaline in the central nervous system lead to depression, urinary frequency and a hyperactive bladder [1]. Alternatively depression may be a result of persistent urinary incontinence, and individuals with altered monoamines in the central nervous system could manifest both depression and an overactive bladder [4]. It is also likely that psychosocial factors can help explain why people with incontinence may become depressed [5].

The prevalence of depression in those experiencing urinary incontinence varies in both clinical and population surveys from 20% to 40% [6-8]. Most studies consider the occurrence of depression and incontinence, without giving consideration to the chronological order or causal pathway of these comorbidities [5-7,9-11]. Some studies determine actual prevalence [7,8], some quote mean scores from depression scales [12], and some suggest a higher risk of depression in those with incontinence [13]. Many studies report the association between incontinence and depression, but venture no further [5,6,9].

Clearly incontinence and depression can affect quality of life (QOL) but only a few studies report this outcome. One population study of women with incontinence found that those with major depression reported significantly lower incontinence-specific quality of life using the I-QOL questionnaire [14]. A second telephone study of women with a mean age of 59 years, reported that major depression predicted the onset of urinary incontinence, but incontinence did not predict the onset of depression [15]. No studies have explored the impact on QOL due to the interaction between incontinence and depression.

This paper examines the QOL in people with urinary incontinence and depression in a population sample of Australian men and women. Our research focuses on psychosocial factors that could explain why people with urinary incontinence get depressed. Potentially this may be a result of incontinence limiting what they are able to do in their everyday lives. We hypothesized that the health related QOL of people with urinary incontinence and depression would be lower than that of people experiencing one of these conditions alone.

## Methods

Data analysed in this study were collected in the 1998 Autumn South Australian Health Omnibus Survey (SAHOS) [16]. SAHOS has investigated a range of health issues since 1990 on an annual basis. It is a representative population survey using a clustered, self-weighting, systematic, multistage area sample of metropolitan and country areas with populations of more than 1000 people and interviews are conducted face-to-face with those aged fifteen years or over. The nature of an omnibus survey means that a number of not necessarily related questions regarding different topics are included from different users. Thus a number of questions not originally intended to be studied together may be analysed to answer a research question.

Data for this survey were weighted by age, sex and geographical location, correcting for any sample bias and providing accurate estimates for the local population overall [17]. The response rate was 70.2% (n = 3010). Questions submitted for SAHOS are reviewed by a management committee. The methodology has been peer reviewed and ethics approval was obtained from the Women’s and Children’s Hospital Human Research Ethics Committee and the South Australian Department of Health Human Research Ethics Committee [16].

In order to determine whether respondents experienced urinary incontinence, they were asked whether they had ever lost any urine when they did not mean to, when they coughed, sneezed or laughed, or if they had ever suddenly felt the urge to go to the toilet, but had accidentally wet themselves before reaching the toilet. Respondents were considered to have urinary incontinence if they answered “yes” to either or both of these questions. These questions reflect the definitions of urinary incontinence used by the International Continence Society (ICS) at the time of the survey, as being “the complaint of any involuntary leakage of urine in the context of type, frequency, severity, precipitating factors, social impact, effect on hygiene and quality of life” [18].

An assessment of depression over the last month was made using the Primary Care Evaluation of Mental Disorders Questionnaire Patient Health Questionnaire (PRIME - MD PHQ) [19]. In this study, the various mental disorders that can be identified with this questionnaire have been collapsed to indicate major depressive syndrome, other depressive syndrome or no depressive syndrome.

The Medical Outcomes Study SF36 was also completed by all respondents in order to assess health related quality of life over the last four weeks. Standard interpretation and scoring methods for the SF-36 were used, and the instrument has been validated for use in an Australian population [20,21].

Demographic information collected for this analysis included gender, age, marital status, household size, country of birth, highest education level achieved, annual household income, work status and area of residence.

Univariate analyses were conducted using SPSS Version 15.0. [22]. Odds ratios and statistical significance (p<0.05) were determined for each demographic subgroup to find which had the highest prevalence of incontinence with depression.

The relationship between a number of variables, incontinence and depression were also explored using multivariate logistic regression analyses. A model was constructed using related variables (p<0.25) In order to determine a model to predict statistically significant urinary incontinence with comorbid depression, related variables (p<0.25) were entered into a logistic regression [23]. Variables determined to be insignificant were progressively omitted until a satisfactory model was obtained. The associations were examined to ensure there were no multicollinearity effects.

For the analysis of health related quality of life, means were generated for each dimension of the SF36 for the following groups: the overall population; those with no incontinence and no depression; those with incontinence only; those with depression only; and those with incontinence and depression. Analysis of variance with a factorial structure (for depression and incontinence) was used to determine whether the mean scores of each of the eight dimensions of the SF36 were significantly different for each of these groups effects using SAS [24] and to determine any interaction.

## Results

### Sample characteristics

Of the n=3010 participants in this study, 48.7% were male and 51.3% were female. The sample is described in Table 1 and these proportions are representative of the sex and age groups of the South Australian population.

**Table 1:** Overall sample demographics

	**Sample demographics**		
**Variable**	**n**	**%**	**95% CI**
**Sex**			
Male	1466	48.7	(46.9–50.5)
Female	1544	51.3	(49.5–53.1)
**Age Group**			
16–39 years	1388	46.1	(44.3–47.9)
40–59 years	1002	33.3	(31.6–35.0)
55 plus years	677	22.5	(21.0–24.0)
**Country of Birth**			
Australia	2267	75.3	(73.7–76.8)
UK/Ireland	382	12.7	(11.5–13.9)
Other	382	12.7	(11.5–13.9)
**Marital Status**			
Married / De facto	1851	61.5	(59.7–63.2)
Separated / Divorced	220	7.3	(6.4–8.3)
Widowed	187	6.2	(5.4–7.1)
Never Married	749	24.9	(23.4–26.5)
**Income**			
Up to $40,000	1484	49.3	(47.5–51.1)
$40,001 to $80,000	834	27.7	(26.1–29.3)
$80,001 plus	247	8.2	(7.3–9.2)
Not stated	445	14.8	(13.6–16.1)
**Overall**	**3010**	**100.0**	

Data Source: South Australian Health Omnibus Survey Autumn 1998

Note The weighting of the data can result in rounding discrepancies or tables not adding.

### Prevalence of urinary incontinence and depression

Table 2 examines the prevalence of urinary incontinence, depression (major or other depressive syndrome) and urinary incontinence with depression by various demographic variables. Urinary incontinence affected 20.3% (n=610) of the study population (male 4.4%, female 35.3%). Female respondents, born in the UK or Ireland, or who were widowed were significantly more likely to experience incontinence when compared with other groups. Those younger than 55 years, with trade or degree qualifications, never married, or a household income of above A$40,000 per annum, were significantly less likely to experience incontinence.

**Table 2:** Univariate analysis of urinary incontinence and depression

	Population with Incontinence*				Population with Depression*				Population with Incontinence and Depression**			
**Variable**	**n**	**%**	**OR (95% CI)**	**p value**	**n**	**%**	**OR (95% CI)**	**p value**	**n**	**%**	**OR (95% CI)**	**p value**
**Sex**												
Male	65/1464	4.4	1.00		194/1464	13.3	1.00		19/65	29.3	1.00	
Female	546/1546	35.3	11.74 (8.97–15.37)	**<0.001**	264/1546	17.1	1.35 (1.11–1.65)	**<0.001**	106/546	19.5	0.58 (0.33–1.03)	0.065
**Age Group**												
55 plus years	272/853	31.9	1.00		118/853	13.9	1.00		50/272	18.4	1.00	
35–54 years	256/1070	23.9	0.67 (0.55–0.82)	**<0.001**	154/1070	14.4	1.04 (0.80–1.35)	0.753	50/256	19.5	1.08 (0.70–1.66)	0.742
16–34 years	83/1087	7.6	0.18 (0.13–0.23)	**<0.001**	186/1087	17.1	1.28 (1.00–1.65)	0.050	25/83	30.7	1.97 (1.13–3.45)	**0.017**
**Area**												
Metropolitan	417/2068	20.1	1.00		324/2068	15.7	1.00		92/417	22.1	1.00	
Country	194/942	20.6	1.03 (0.85–1.25)	0.758	134/942	14.2	0.89 (0.72–1.11)	0.742	33/194	17.2	0.73 (0.47–1.14)	0.163
**Education**												
No post school education	397/1682	23.6	1.00		298/1682	17.7	1.00		90/397	22.6	1.00	
Trade Qualifications	28/373	7.5	0.26 (0.17–0.39)	**<0.001**	53/373	14.1	0.76 (0.56–1.05)	0.096	6/28	21.5	0.94 (0.37–2.38)	0.889
Certificate/Diploma	131/599	21.8	0.90 (0.72–1.13)	0.373	74/599	12.4	0.66 (0.50–0.87)	**0.003**	24/131	18.1	0.76 (0.46–1.25)	0.280
Degree or higher	55/356	15.4	0.59 (0.43–0.80)	**0.001**	33/356	9.3	0.48 (0.33–0.70)	**<0.001**	6/55	10.6	0.41 (0.17–0.99)	**0.047**
**Country of Birth**												
Australia	439/2266	19.4	1.00		340/2266	15.0	1.00		88/439	20.0	1.00	
UK/Ireland	91/381	23.9	1.31 (1.01–1.69)	**0.042**	58/381	15.3	1.02 (0.76–1.38)	0.889	21/91	22.9	1.19 (0.69–2.05)	0.524
Other	81/363	22.2	1.19 (0.91–1.55)	0.209	60/363	16.5	1.11 (0.83–1.51)	0.478	17/81	20.6	1.04 (0.58–1.87)	0.894
**Marital Status**												
Married / De facto	439/1851	23.7	1.00		248/1851	13.4	1.00		78/439	17.7	1.00	
Separated / Divorced	57/221	26.0	1.13 (0.82–1.55)	0.453	52/221	23.7	2.01 (1.43–2.82)	**<0.001**	14/57	24.2	1.49 (0.77–2.85)	0.233
Widowed	73/187	39.2	2.07 (1.52–2.83)	**<0.001**	26/187	14.1	1.06 (0.69–1.64)	0.783	17/73	23.4	1.42 (0.78–2.57)	0.249
Never Married	40/748	5.4	0.18 (0.13–0.26)	**<0.001**	129/748	17.3	1.35 (1.07–1.70)	**0.011**	16/40	39.5	3.03 (1.54–5.97)	**<0.001**
**Income**												
Up to $40,000	357/1484	24.1	1.00		284/1484	19.2	1.00		96/357	26.8	1.00	
$40,001 to $80,000	132/834	15.8	0.59 (0.48–0.74)	**<0.001**	96/834	11.5	0.55 (0.43–0.70)	**<0.001**	12/132	9.2	0.28 (0.15–0.52)	**<0.001**
$80,001 plus	40/247	16.1	0.61 (0.42–0.87)	**0.006**	22/247	9.1	0.42 (0.27–0.66)	**<0.001**	6/40	16.3	0.53 (0.22–1.27)	0.155
Not stated	82/444	18.4	0.71 (0.55–0.93)	**0.013**	56/444	12.6	0.61 (0.45–0.83)	**0.002**	11/82	13.4	0.42 (0.21–0.83)	**0.012**
**Overall**	**610/3010**	**20.3**			**459/3010**	**15.2**			**125/610**	**20.5**		

Data Source: South Australian Health Omnibus Survey Autumn 1998.

Note The weighting of the data can result in rounding discrepancies or tables not adding.

*Of the total population.

**Of those with Incontinence.

Respondents with a major (6.7%) or other depressive (8.6%) syndrome made up 15.2% (n=459) of the study population (male 13.3%, female 17.1%). Females, those separated or divorced, or never married were more likely to experience depression compared to other groups, whereas those with a certificate or diploma or degree or higher, or with a household income greater than A$40,000 per annum were less likely to experience depression.

Overall it was found that 4.3% of the population experienced urinary incontinence with comorbid depression. There was a statistically significant higher rate of major or other depressive syndrome in the urinary incontinent (20.5% [n=125/610]) compared with those without urinary incontinence (13.9% [n=333/2399]). Of these respondents with urinary incontinence, 29.3% of males and 19.5% of females experienced a major or other depressive syndrome. It was found that those aged 16 to 34 years and never married were significantly more likely to experience depression if they also had urinary incontinence, whereas those with a bachelor’s degree or higher, a household income of A$40,001 to A$80,000 per annum or did not state their income, were significantly less likely to experience depression if they were urinary incontinent.

Multivariate analysis showed that variables jointly identified as increasing the risk urinary incontinence with depression were those with Fair or Poor overall health and those who thought that their urinary incontinence was moderately or very serious. Respondents who had a household income between A$40,001 and A$80,000 per annum or did not state their income, were not current smokers, and had a lifetime occupation of being a tradesperson were less likely to have incontinence with depression (model ×^2^ = 167.22, df = 53, p <0.001) (Table 3).

**Table 3:** Multivariate analysis of variables which determined incontinence with co-morbid depression

**Variables**	**n**	**%**	**OR (95% CI)**	**p value**
**Overall health status**				
Excellent	9/106	8.7	***1.00***	
Very Good	24/198	12.3	1.48 (0.61–3.62)	0.385
Good	20/154	13.2	1.32 (0.51–3.38)	0.568
Fair	51/116	44.1	9.84 (3.80–25.48)	**<0.001**
Poor	20/37	54.6	12.74 (3.78–42.95)	**<0.001**
**Income**				
Up to $40,000	96/357	26.8	***1.00***	
$40,001 to $80,000	12/132	9.2	0.30 (0.14–0.68)	**0.004**
$80,001 plus	6/40	16.3	0.38 (0.12–1.22)	0.106
Not stated	11/82	13.3	0.41 (0.18–0.95)	**0.037**
**Smoking status**				
Current smoker	40/115	34.5	***1.00***	
Ex smoker	32/188	16.8	0.46 (0.23–0.95)	**0.035**
Non smoker	54/308	17.5	0.46 (0.24–0.89)	**0.021**
**Lifetime Occupation**				
Not employed	31/119	26.3	***1.00***	
Managers & Administrators	6/33	18.3	0.70 (0.21–2.33)	0.561
Professionals	9/56	15.3	1.00 (0.29–3.47)	0.995
Para-Professionals	5/39	12.8	0.34 (0.08–1.40)	0.135
Tradespersons	7/42	16.9	0.25 (0.07–0.90)	**0.034**
Clerks	23/141	16.1	0.82 (0.37–1.84)	0.636
Sales or Service workers	19/87	21.8	1.01 (0.43–2.36)	0.989
Drivers & Machine Operators	4/16^#^	23.9	--	--
Labourers	22/76	28.2	0.65 (0.26–1.58)	0.337
Not stated	0/1^#^			
**How serious**				
Not very, not serious, refused	92/504	18.3	***1.00***	
Very/moderately serious	33/102	32.6	2.30 (1.20–4.41)	**0.012**
**Overall**	125/610	20.5		

Data Source: South Australian Health Omnibus Survey Autumn 1998.

Note The weighting of the data can result in rounding discrepancies or tables not adding.

# Numbers too small for statistical analysis.

### UI, depression and quality of life

Health-related quality of life was assessed for people with different combinations of urinary incontinence and depression. Groups that were mutually exclusive were compared using analysis of variance for significant differences. Mean scores adjusted for age and sex for each of the eight dimensions of the SF-36 scale were calculated and results are presented in Table 4.

**Table 4:** SF36 Mean Scores for people with urinary incontinence, depression and combinations of these conditions (adjusted for age and sex)

	**n**	**PF**	**RP**	**BP**	**GH**	**VT**	**SF**	**RE**	**MH**
No Incontinence and No Depression	2066	88.07	84.93	75.55	77.92	69.39	92.67	95.16	85.04
***General Population***	*3010*	***85.31***	***79.82***	***72.54***	***73.91***	***64.35***	***87.90***	***87.83***	***79.99***
Incontinence without Depression	486	85.00^aa^	78.58^aa^	72.72^aa^	74.25^aa^	65.46^aa^	91.10	91.53^aa^	82.40^aa^
Depression without Incontinence	333	77.49^aabb^	64.29^aabb^	61.84^abb^	60.13^ab^	43.90^ab^	67.56^ab^	58.12^ab^	58.37^ab^
Incontinence with Depression	125	66.33^abc^	49.88^abcc^	56.11^ab^	50.60^abcc^	40.94^ab^	61.41^ab^	46.72^abcc^	55.28^ab^
p-value for interaction term		0.0002	0.046	0.27	0.02	0.54	0.09	0.02	0.97

^a^ Statistically significantly lower (*t* test p<0.001) than those with no incontinence and no depression.

^aa^ Statistically significantly lower (*t* test p<0.05) than those with no incontinence and no depression.

^b^ Statistically significantly lower (*t* test p<0.001) than those with incontinence but no depression.

^bb^ Statistically significantly lower (*t* test p<0.05) than those with incontinence but no depression.

^c^ Statistically significantly lower (*t* test p<0.001) than those with depression but no incontinence.

^cc^ Statistically significantly lower (*t* test p<0.05) than those with depression but no incontinence.

Respondents who reported that they experienced urinary incontinence with depression scored significantly lower than those experiencing neither urinary incontinence nor depression, and also with those with urinary incontinence but no depression, on all dimensions of the SF-36 (Physical Functioning (PF), Role Physical (RP), Bodily Pain (BP), General Health (GH) (Vitality (VT), Mental Health (MH), Social Functioning (SF), Role Emotional (RE) (p < 0.05)). Additionally this group scored significantly lower on most dimensions than those with depression only (PF (p < 0.001); RP, GH, RE (p < 0.05)).

Overall, respondents with depression only, scored significantly lower across all dimensions of the SF-36 (PF, RP, (p > 0.05) BP, GH, VT, RE, MH (p<0.001)), when compared with those respondents who had no depression and no urinary incontinence, and significantly lower than those with urinary incontinence only (PF, RP, BP (p > 0.05), GH, VT, SF, RE, MH (p<0.001)).

Those respondents with incontinence only, scored significantly lower across most dimensions of the SF-36 except for Social Functioning (PF, RP, BP, GH, VT, RE, MH (p > 0.05)), when compared with those respondents who had no depression and no urinary incontinence.

The interaction term was statistically significant for PF, RP, GH, and RE. For BP, VT, SF and MH the main effect for depression and the main effect for urinary incontinence were both statistically significant. For ease of interpretation the interaction means for all standardized scores are presented in Table 4.

**Figure 1:**
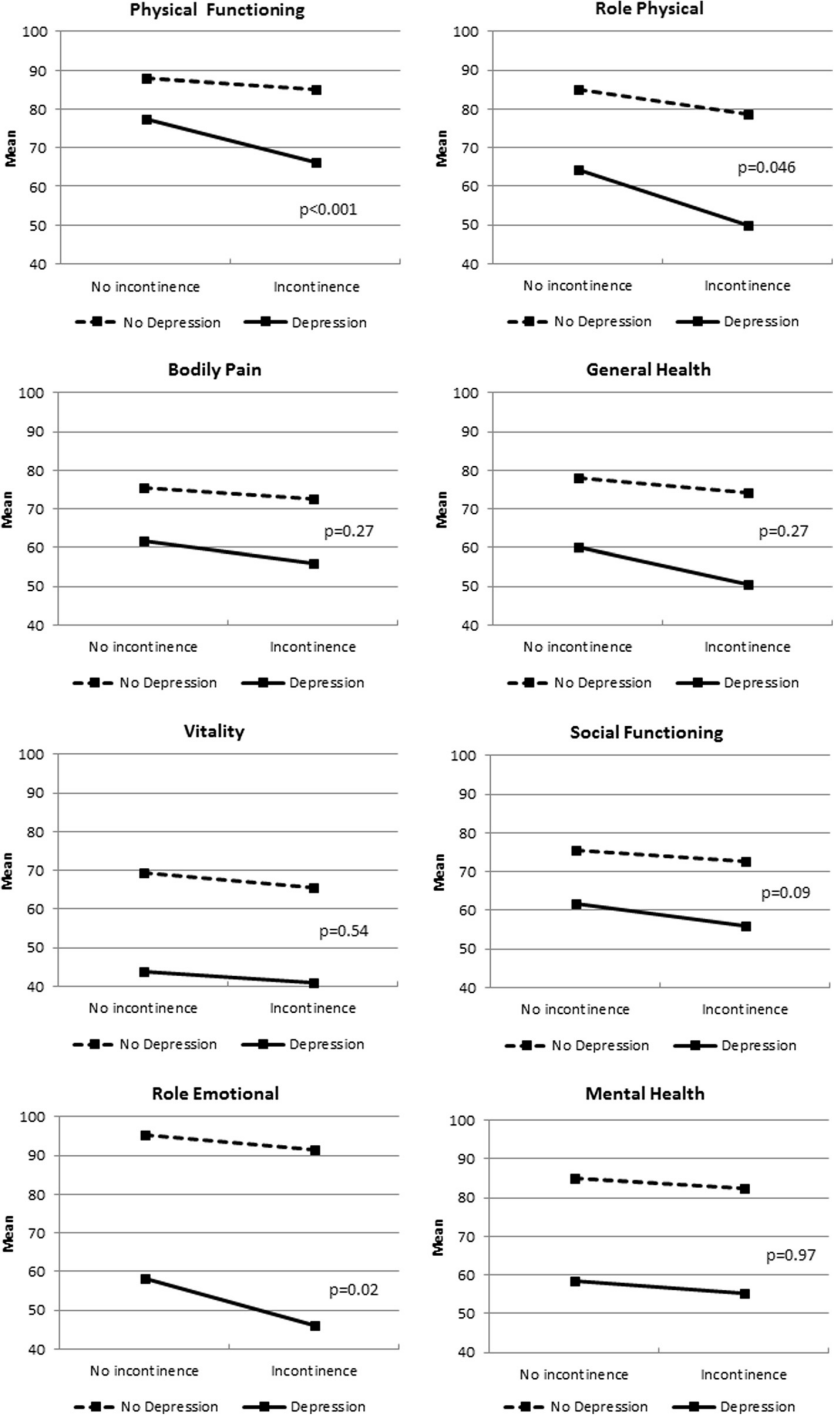
Quality of Life interaction plots for people with and without Incontinence, and with and without Depression (adjusted for age and sex).

For each of the standardized scores, the mean score for each combination of depression and urinary incontinence is presented graphically in an interaction plot (Figure 1). The lines drawn between the means allow visual interpretation of the interactions.

The effect of depression results in a much greater reduction in mean score for both the not incontinent group and the incontinent group. However, the significance of the interaction (for PF, RP, GH and RE) is most likely due to those who have both depression and urinary incontinence having a significantly greater reduction in score, compared to those with depression who are not urinary incontinent. Although this reduction in mean score was observed for the other SF36 score variables (BP, VT, SF and MH) also, the difference was not large enough to be statistically significant.

## Discussion

In this face to face survey of 3010 South Australians self-reported urinary incontinence was found in 20.3% (n=610), depression in 15.2% (n=459) and both in 4.3% of respondents. Those with urinary incontinence and comorbid depression were more likely to be aged between 15 to 34 years and never married when compared to those with only incontinence. Multivariate analysis demonstrated that in those with urinary incontinence, an overall health status of Fair or Poor, or the perception that their incontinence was moderately or very serious, increased the risk of having comorbid depression. Depression had a marked effect on QOL for the general population and a significant, additive effect on those with incontinence. Respondents who reported that they experienced urinary incontinence with comorbid depression scored significantly lower than those experiencing incontinence without depression on all dimensions of the SF-36. The interaction between urinary incontinence and depression had a significant effect on the physical functioning dimensions of quality of life.

The quality of life of people who experience urinary incontinence with depression, in both adult females and males of all age groups, has not been assessed previously via population surveys using face to face interviews. Other studies have assessed this qualitatively, or have discussed stigma, and other problems associated with incontinence including depression. But how urinary incontinence and depression interact and affect QOL has not been considered [25-28]. A lack of population data prompted the retrospective analysis of an existing dataset, already available from the 1998 SAHOS, where questions regarding urinary incontinence, depression and quality of life were asked together. At the time of this study, the questions about urinary incontinence were not validated, however they reflected the definition used by the International Continence Society (ICS) [18]. They have since been validated by other authors [29].

This study has several limitations. Firstly the symptoms of urinary incontinence were not clinically quantified. However, in a population study of this size, it would not be practical to clinically examine cases for this condition, and prevalence rates using self-report have been found to be similar and cost less compared to those found from diagnostic tests [30]. Secondly because recall times differ for urinary incontinence, depression and the quality of life measures, it is possible, that depression and urinary incontinence did not co-exist when the survey was administered. However urinary incontinence and depression are relapsing and remitting conditions and it is difficult to examine the temporality and causality in a cross sectional study. Lastly the use of the PRIME MD in this study to determine depression deviates slightly from the original intentions of its authors [19], as the initial depression screening questions were not used, and the mood module was administered to all in the study. However the prevalences of urinary incontinence (20.3%) [31] and major (6.7%) or other (8.6%) depressive syndrome (15.2%) [32] are comparable with other studies. Circumstances where both these conditions occur together (20.5% of those with urinary incontinence) are also equivalent to international studies [3,7].

Univariate analysis indicates that younger people, and those never married were more likely to experience depression when they had urinary incontinence. This is not unexpected, as incontinence is often considered a disease of older women who have had children, possibly a plausible explanation for their incontinence. In the above group, there may not be an explanation for the condition, leading to a state of low mood and depression.

In the multivariate analysis, self-reported Fair or Poor health, and the perception that one’s own urinary incontinence was moderately or very serious were strongly predictive of having incontinence with depression. This may indicate that one’s own perceptions of a condition, and their overall health may lead to an increased likelihood of experiencing mental illness. However as this study was cross sectional, we were unable to determine whether the depression was caused by incontinence, or a person’s depression increased their perception of symptom severity. This will be explored in future qualitative work.

In the quality of life analysis, we compared respondents with “Incontinence with depression” to those with “Incontinence without depression”. “Incontinence with depression” describes respondents who answered in the positive for any of the incontinence questions, and includes those who also scored positively for depression by the PRIME-MD. “Incontinence without depression” includes respondents with urinary incontinence, not diagnosed with depression by the PRIME-MD in this survey. Respondents with urinary incontinence and depression scored significantly lower on all dimensions of the SF 36, with depression scoring lower than urinary incontinence and those with both conditions together scoring lowest of all. When these conditions occur together, there was a major additive effect particularly in the Mental Health scales, greater than that with either condition alone. It appears that depression increases a person’s negative perceptions of their physical symptoms (incontinence) reducing their QOL scores further than would be expected if either condition occurred independently. This effect is also reflected in the interaction between incontinence and depression and its impact on the QOL dimensions that measure physical functioning.

It may be that identifying and treating depression in a person with urinary incontinence, a patient’s mental health (QOL) will not only improve but also, indirectly their physical QOL.

## Conclusions

Depression and urinary incontinence both reduce QOL. When they occur together there appears to be an additive effect which affects both physical and mental health. Clinicians should identify and manage comorbid depression when treating patients who have incontinence to improve their overall QOL.

## Competing interests

The authors declare that they have no competing interests.

## Authors’ contributions

JCA drafted the manuscript, NS is her primary PhD supervisor, and PD, ABM and AT are co supervisors. AT also manages the SAHOS survey where the data from this study originated and also had editorial input into the paper, RDG and AHM are the original owners of the data, formulating the original questions regarding depression and urinary incontinence in this survey. All seven authors edited and approved the paper.

## Author’s information

JCA is a candidate for a PhD in Medicine.

## Pre-publication history

The pre-publication history for this paper can be accessed here:

http://www.biomedcentral.com/1471-2490/13/11/prepub
